# Pivotal models and biomarkers related to the prognosis of breast cancer based on the immune cell interaction network

**DOI:** 10.1038/s41598-022-17857-x

**Published:** 2022-08-11

**Authors:** Rui Liu, Xin Yang, Yuhang Quan, Yiyin Tang, Yafang Lai, Maohua Wang, Anhao Wu

**Affiliations:** 1 Department of Mammary Surgery I, The Third Affiliated Hospital of Kunming Medical University (Yunnan Cancer Hospital, Yunnan Cancer Center), No. 519, Kunzhou Road, Kunming, 650118 China; 2 Department of Blood Transfusion, The First People’s Hospital of Yunnan Province, The Affiliated Hospital of Kunming University of Science and Technology, Kunming, China; 3 Department of Anesthesiology, The Third Affiliated Hospital of Kunming Medical University (Yunnan Cancer Hospital, Yunnan Cancer Center), Kunming, China; 4 Department of Mammary Surgery II, The Third Affiliated Hospital of Kunming Medical University (Yunnan Cancer Hospital, Yunnan Cancer Center), Kunming, China; 5 Kunming Women and Child Health Service Center/Kunming Women and Child Health Care Hospital, Kunming, China

**Keywords:** Breast cancer, Biomarkers

## Abstract

The effect of breast cancer heterogeneity on prognosis of patients is still unclear, especially the role of immune cells in prognosis of breast cancer. In this study, single cell transcriptome sequencing data of breast cancer were used to analyze the relationship between breast cancer heterogeneity and prognosis. In this study, 14 cell clusters were identified in two single-cell datasets (GSE75688 and G118389). Proportion analysis of immune cells showed that NK cells were significantly aggregated in triple negative breast cancer, and the proportion of macrophages was significantly increased in primary breast cancer, while B cells, T cells, and neutrophils may be involved in the metastasis of breast cancer. The results of ligand receptor interaction network revealed that macrophages and DC cells were the most frequently interacting cells with other cells in breast cancer. The results of WGCNA analysis suggested that the MEblue module is most relevant to the overall survival time of triple negative breast cancer. Twenty-four prognostic genes in the blue module were identified by univariate Cox regression analysis and KM survival analysis. Multivariate regression analysis combined with risk analysis was used to analyze 24 prognostic genes to construct a prognostic model. The verification result of our prognostic model showed that there were significant differences in the expression of PCDH12, SLIT3, ACVRL1, and DLL4 genes between the high-risk group and the low-risk group, which can be used as prognostic biomarkers.

## Introduction

Breast cancer ranks the first in the incidence of female cancer, and maintains an upward trend year by year^1^. Globally, about 2.1 million cases of female breast cancer were newly diagnosed in 2018, accounting for nearly a quarter of cancer cases in women^2^. Although advances in early diagnosis and comprehensive treatment strategies have obviously enhanced the prognosis of breast cancer patients in recent years, nearly 30% breast cancer patients still develop metastases after diagnosis and treatment. Here, it should be noted that the 5-year overall survival rate for patients with non-metastatic breast cancer was greater than 80%, while the survival rate for patients with metastatic breast cancer was less than 30%^3,4^.

Although the expression of the estrogen receptor (ER), the progesterone receptor (PR) and the ERBB2 receptor (HER2) has laid the foundation for the classification of breast cancer, breast cancer is divided into at least five molecular subtypes (i.e., Luminal A, Luminal B, Her2-enriched, Basal-like and Normal-like) based on gene expression. However, as research progresses, the genomic/transcriptome level of breast cancer typing keeps sustained growth^5^. These studies confirm the heterogeneity of breast cancer, which is also reportedly one of the leading causes of breast cancer treatment failure, recurrence, and patient death^6^. Tumor heterogeneity not only leads to distinctions in survival and prognosis of disparate patients, but also brings about different biologic characteristics of cancer cells and different responses to chemotherapy drugs^7^.

While the heterogeneity of breast cancer has been found and confirmed, the existence of different molecular subtypes of breast cancer and different cell subsets within the same tumor tissue also needs to be taken into account in the implementation of individualized treatment of breast cancer^8^. However, the biological relationships between different clonal subsets and between clones and microenvironment in breast cancer tissues are still unclear. Traditional gene sequencing methods can only detect population cells but fail to reflect genetic traits at the single-cell level. Single-cell sequencing technology is conducive to studying tumor heterogeneity from differences at the single-cell level and facilitating the comparison of differences between different subtypes of the same tumor^9^. In this study, single-cell sequencing data was used to identify the inter-tumor and intra-tumoral heterogeneity of breast cancer samples. By the identification of the characteristic genes of immune cell subtypes and the combination with known immune cell marker genes, a multi-factor interaction network of receptor-ligand-transcription factors in breast cancer was constructed. WGCNA was employed to identify the prognostic signature, and a prognostic model was constructed for evaluation and verification.

## Materials and methods

### Data sources and processing

Two sets of data, GSE75688 and GSE118389, were downloaded from GEO, among which GSE75688 contained single cell sequencing data (sRNA-seq) of primary breast cancer and metastatic breast cancer, and GSE118389 was sRNA-seq data of triple negative breast cancer. The RNA sequencing data of gene expression (FPKM value) and clinical information are downloaded from UCSC Xena (https://gdc.xenahubs.net). The data of TCGA-BRCA are processed in the following steps: (1) Remove the samples without clinical follow-up information; (2) Remove samples with unknown survival time, less than 0 days and no survival status; (3) Turn the probe into gene symbol; (4) If one probe corresponds to multiple genes, and the probe is removed; (5) Take the median value if the expression with multiple gene symbols. The Create Seurat Object function is applied to process the Seurat object. After two sets of data were analyzed by PCA, the Find Integration Anchors function is used to integrate the two sets of data in the S4 object. Finally, the data splits into three groups: primary breast cancer, metastatic breast cancer, and triple negative breast cancer.

### PCA dimension reduction, cell clustering and annotation

The integrated data is preprocessed using the Seurat package in R. After PCA dimension reduction, JackstrawPlot and ElbowPlot are used to show the overall situation of the data. The default value of K being 20 and the resolution being 0.2. According to experience and debugging, 0.2 is selected as the threshold value for cell clustering, and 14 cell clusters are obtained. Subsequently, marker genes in the Cell marker and Panglao DB databases and genes reported in the literature were utilized to annotate the cell clusters^10,11^. The Find All Markers function in the Seurat package was used for differential analysis of single-cell data.min.pct = 0.25, only. pos = TRUE, leave the rest of the fields to their default. According to the expression levels of top 5 genes and marker genes of immune cell reported in the literature, marker genes were displayed by the dotplot and violin plots in each cluster. In addition, the dotplot was used to show the proportion of immune cells in different groups in line with the frequencies of individual cells in the Primary BC group, the Metastatic BC group and the TNBC group.

### Construction of ligand-receptor network and joint analysis of transcription factors

In this section, The c2.cp.kegg.v7.2.symbols.gmt gene set is obtained from the molecular signature database v7.2 (https://www.gsea-msigdb.org/gsea/downloads.jsp) download page. The cellphoneDB software (https://github.com/Teichlab/cellphonedb) is adopted. In order to study the potential interactions between different cell types in TME, CellPhoneBD is used for intercellular communication analysis. CellPhoneBD is a publicly available repository of selected receptors, ligands and their interactions. CellPhoneBD analysis is carried out using the CellPhoneBD Python package (2.1.7). After the software was downloaded to build a favorable environment, according to the code cellphoneDB method statistical analysis meta.txt counts.txt, the interaction between ligands and receptors in each group of data is analyzed^12^. Among them, the meta.txt file is the barcode and corresponding annotated cells; counts.txt denotes the barcode and the gene expression matrix. In the results, P values of cell types and enriched interaction ligands and receptors were shown. With P < 0.05 as the threshold, cellphoneDB is used to plot the heatmap and meta.txt p values. For further analysis of the transcription factor regulation of ligands and receptors, the database TRRUST (https://www.grnpedia.org/trrust/) is adopted, and the hypergeometric test method is used to trace the transcription factors of the target ligand and receptor genes, and Cytoscape 3.7.2 is applied in the visual display of results.

### Weighted gene co-expression network analysis

Considering that WGCNA is a systematic biology approach to construct scale-free networks using gene expression data, the WGCNA package of R was used to construct a weighted co-expression network in the light of the expression profile data of the multifactorial network genes^13^. Initially, the expression level of the transcript was transformed into a similarity matrix based on the Pearson correlation between paired genes. Then, the similarity matrix is transformed into adjacency matrix. β parameters can enhance the strong correlation between genes and lower the weak correlation between genes. When the power of β is 18, the adjacency matrix is transformed into a topological overlap matrix. To classify genes with similar expression patterns into different modules, a dynamic hybrid cutting method is adopted. Meanwhile, the minimum number of genes in the module is truncated to 30. KEGG pathway enrichment is a common analytical method in bioinformatics to understand the role of genes in biological systems. Metascape (http://metascape.org) is utilized to perform functional enrichment analysis, which is an online analysis tool that integrates several ontology sources, including the KEGG pathway, GO biological processes, canonical pathways, and CORUM. Significant pathways were screened according to P < 0.05. The prognostic modules were screened in accordance with characteristics of overall survival time and overall survival status. The survival time is the total survival time of each patient, which is the contact value. The survival status is 0 and 1, which refers to classification variables. 0 means survival while 1 represents death. GO enrichment analysis was performed on each module by Metascape to analyze the biological functions of each module. Moreover, as the first software to use the method of hypergeometric distribution to determine the significance of pathway enrichment, KOBAS has been successfully applied to the study of different organisms, such as plants, animals and bacteria. The KOBAS server can be accessed via https://kobas.cbi.pku.edu.cn. KOBAS is used for KEGG pathway enrichment analysis in this paper, and P < 0.05 is deemed to be of significance. So the website KOBAS is employed to perform KEGG Pathway enrichment analysis of co-expressed genes in modules related to the prognosis of breast cancer.

### Univariate regression analysis, KM-survival prognostic analysis and multivariate regression analysis

First, Univariate Cox regression analysis was used to screen genes significantly expressed in key modules. Then, genes with striking differences in the univariate regression analysis were analyzed by Lasso dimensionality reduction, output results of which were used as candidate genes. Furthermore, K-M survival analysis was used to identify prognostic related genes, and P < 0.05 was used as a threshold to screen genes with prominent prognostic effects. The candidate genes and prognostic genes were intersected and visualized by the Venn diagram. In this study, 24 genes associated with prognosis of breast cancer were identified by univariate Cox regression analysis, and results of univariate cox regression for 24 genes are displayed by forest maps. The survminer and survival packages are used to perform multivariate regression analysis. Age, lymphatic node metastatic status (N0 vs NN (excluding NX)), the T stage, radiotherapy, race, the breast cancer stage, and overall survival were combined with 24 genes for multivariate regression analysis to identify pivotal genes bound up with breast cancer prognosis.

The breast cancer samples were segmented into high-risk and low-risk groups based on the median expression value of the screened key genes relevant to breast cancer progression. In terms of time-dependent ROC results, the AUC of the combined Signature 3-year model group was 0.801 for the analysis of immune infiltration levels in the low-risk group. The time-dependent ROC is used to reflect the accuracy and precision of the prediction model. It is generally believed that the model with AUC >  = 0.7 can be conducive to the prediction of the prognostic outcome at a specific time^14,15^.

## Results

### Quality control, dimensionality reduction, cell clustering and annotation of breast cancer single cell sequencing data

In this study, two sets of data (GSE75688 and GSE118389) were downloaded. GSE75688 contains single-cell RNA sequencing data of primary and metastatic breast cancer, with a total of 563 cells. Among them, 12 samples were sequenced in bulk. Except for non-single cell sequencing data, 549 single cell data were retained, among which 441 cells are primary breast cancer and 108 cells are metastatic breast cancer. GSE118389 is the scRNA sequencing data of triple-negative breast cancer containing 1534 cells. In the quality control of single-cell transcriptome data, the number of feature genes is greater than 200 and less than 2500, and the proportion of mitochondrial (percent.mt) less than 10% is used as a threshold for data screening and filtering. The first threshold is set to eliminate the empty oil droplets. To avert a low number of RNA, data less than 200 is eliminated. The second threshold is set to eliminate more than two cells into one oil droplet. Subsequently, PCA and UMAP dimensionality reduction are performed on the data, and the results are visualized in the form of the heat map, the JackstrawPlot, and the ElbowPlot (Supplementary Fig. 1).

**Figure 1:**
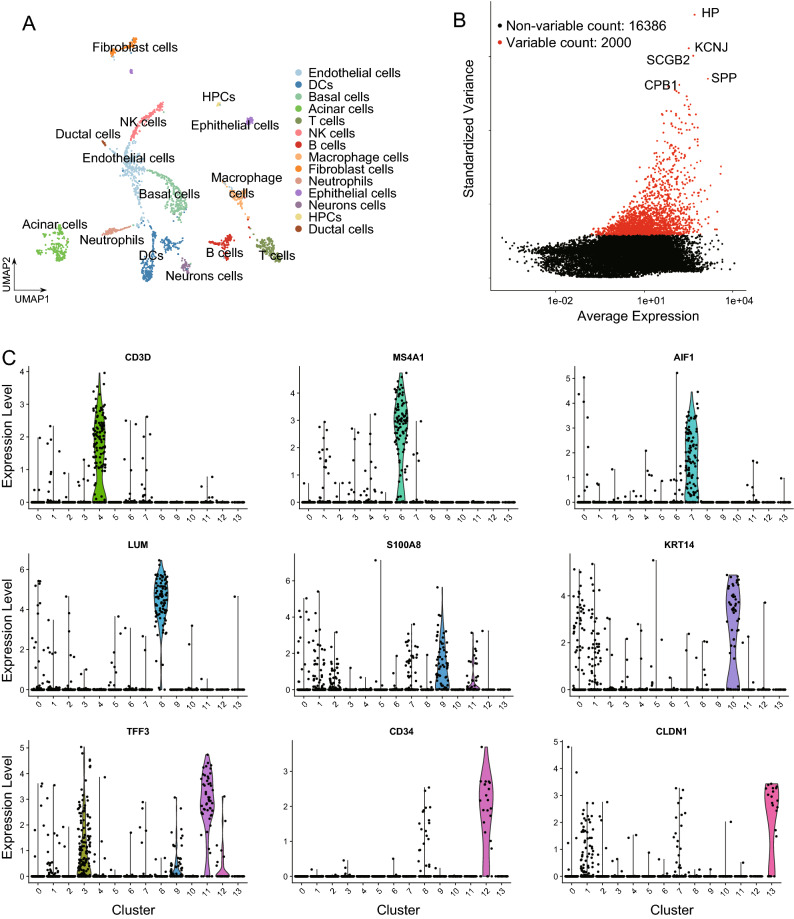
Single-cell clusters of breast cancer and the expression of marker genes in each cluster. (**A**) Visualization of cell UMAP clustering results. (**B**) Visualization of the genes with the highest differential variance and non-differential genes. (**C**) The violin chart shows the expression of common cell marker genes in the corresponding cell clusters.

Next, the Find Integration Anchors function is used to integrate two single-cell transcriptome sequencing data from two data sets (non-merged, since the merged one is only A data merge unable to remove batch effect), which minimizes the error caused by different batches of experiments, and is thus used to construct the final S4 object. Subsequently, the Scale Data was used for data centralization and standardization, and Seurat continued to be used for PCA and UMAP dimensionality reduction analysis. According to references and debugging effects, the clustering analysis were carried out with a threshold of 0.2 resolution, and 14 clusters were acquired. The cell clusters were annotated on the basis of the marker genes in the Cellmarker, the PanglaoDB database and references. The results showed that 14 clusters were endothelial cells, DCs, basal cells, acinar cells, T cells, NK cells, B cells, macrophage cells, Fibroblast cells, neutrophils, epithelial cells, neurons cells, HPCs, and ductal cells (Fig. 1A, Supplementary Table 1). The Find Variable Features function is used to find the genes that differ the most from one to another among cell clusters. The results show that HP, KCNJ, SCGB2, CPB1 and SPP are the first five significantly different genes (Fig. 1B). Meanwhile, expression of common cell markers, namely CD3D, MS4A1, AIF1, LUM, S100A8, KRT14, TFF3, CD34 and CLDN1, in 14 clusters were analyzed, and the Violin map was used to show the results of marker genes in each cluster (Fig. 1C).

### Differential gene expression and multi-factor interaction analysis of immune cells in breast cancer

**Figure 2:**
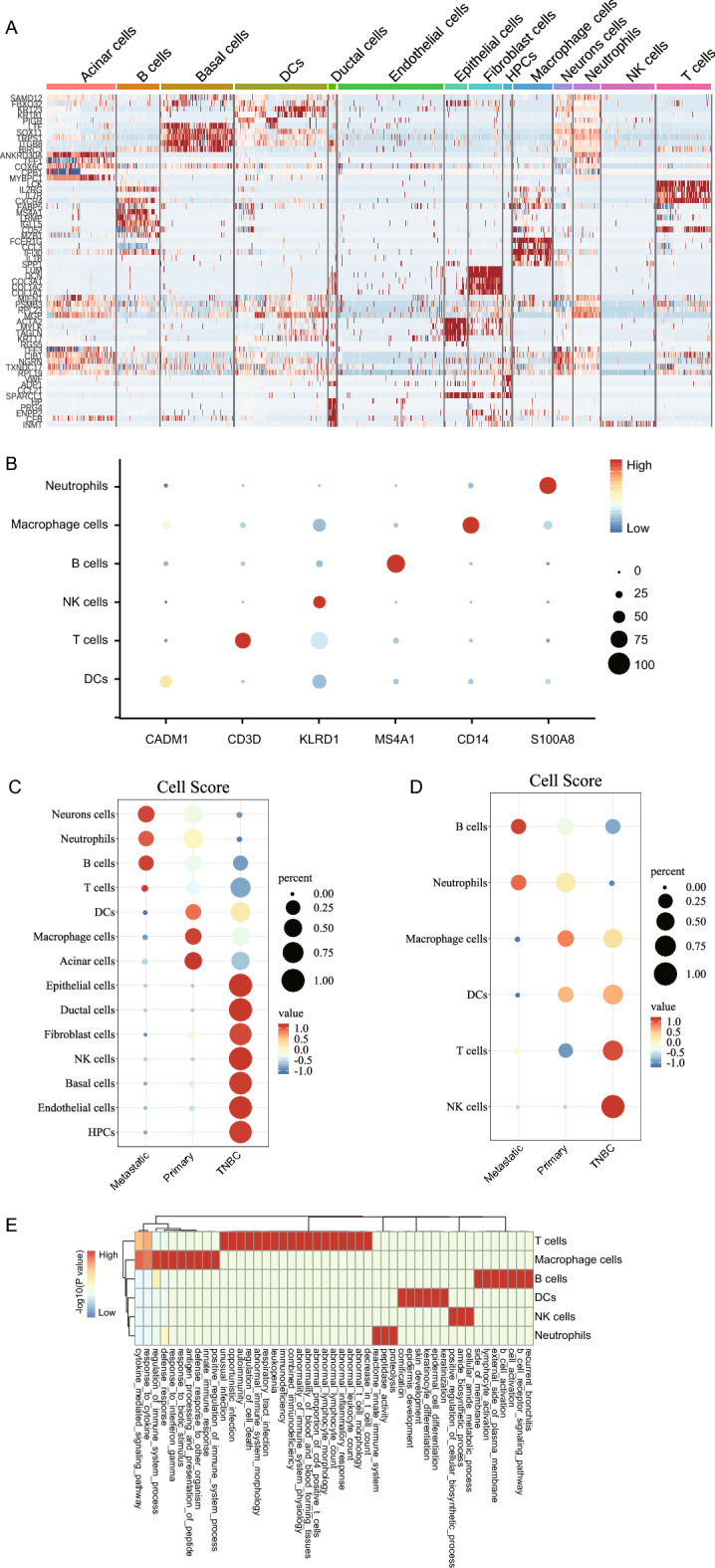
Differentially expressed genes, immune marker genes and cell ratio enrichment analysis. (**A**) The differential gene heat map of disparate cell types. (**B**) Analysis of immune marker gene expression. (**C**) Cell ratio enrichment analysis of 14 cell clusters. (**D**) Enrichment analysis of the immune cell ratio. (**E**) Functional annotation analysis of immune cells.

In order to analyze the differentially expressed genes in each cluster, the Find All Markers function is used to calculate the expression of differential genes in each cluster, and the Do Heatmap function to plot the distribution of differential genes in different cell types (Fig. 2A). According to the conventional genes of cell annotation in the literature, the expression of 6 types of immune cell marker genes, including NK, DCs, macrophages, B cells, T cells and neutrophils, was analyzed (Fig. 2B, Supplementary Table 2). Subsequently, the frequency of cells in each cluster is counted and used to explore the enrichment ratio of immune cell populations. The results show that NK cells are significantly aggregated in triple-negative breast cancer; the proportion of macrophages is remarkably increased in primary breast cancer; B cells, T cells and neutrophils may play a vital role in metastatic breast cancer (Fig. 2C,D). As exciting as this sounds, T cells and neutrophils are reported to be involved in metastasis of breast cancer^16^. Next, the MSigDB database is used to perform functional annotation analysis of cell types, which is conducive to revealing the functional status of immune cells. The analysis results showed that the functions of specifically expressed genes in T cells, Macrophage cells, B cells, DC cells, and Neutrophils were dramatically enriched in 20, 10, 7, 6, 3, and 3 terms, respectively (Fig. 2E).

**Figure 3:**
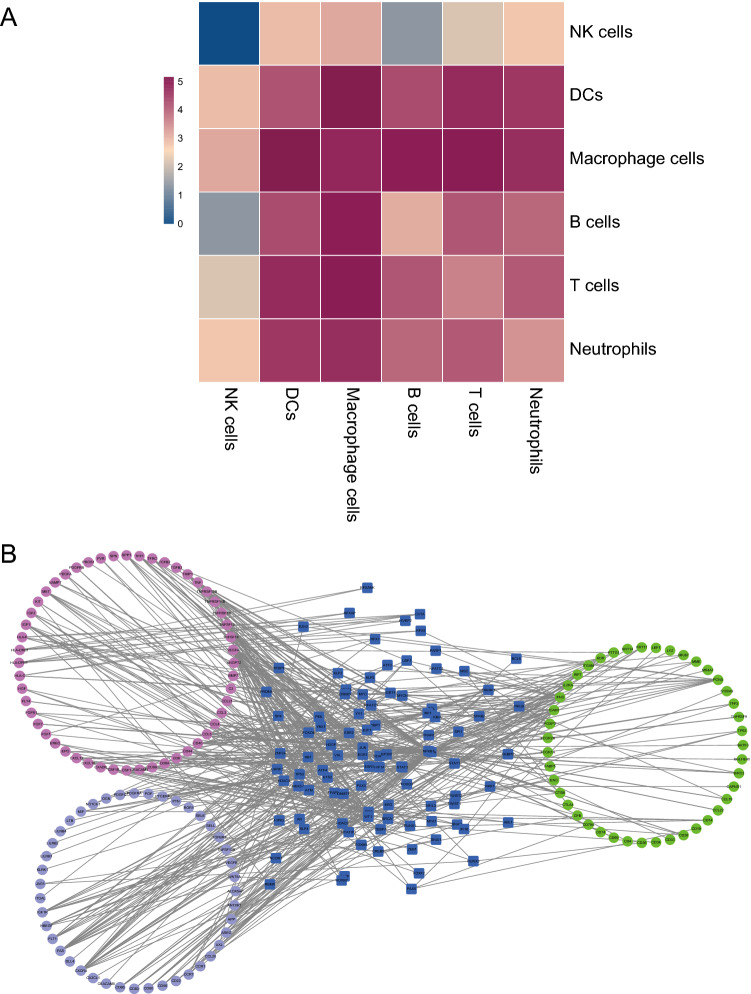
CellphoneDB analysis of cell–cell interaction. (**A**) The heat map of cell–cell interaction on the strength of the ligand-receptor. (**B**) The multi-factor interaction network of ligand-receptor in combination with the transcription factor (Blue represents the transcription factor TF; green denotes the immune marker gene; red represents the ligand gene ligand, and light blue represents the receptor gene receptor).

On the basis of elucidating the effect of differential gene expression in breast cancer samples on immune cells, the ligand-receptor relationship between cells is analyzed by the cellphoneDB software. In the output of the ligand receptor results (Supplementary Table 3), the interaction between immune cells is analyzed using the heatmap plot function. The color-coded values are log10 (the count of interactions between the ligand and the receptor). The results showed that macrophages and dendritic cells have obvious activity and interact with a variety of cells (Fig. 3A). In order to further analyze the interaction between the ligand and the receptor, the TRRUST database was applied, and the hypergeometric test method was used to analyze the interaction between differential genes, immune cell marker genes, and ligand-receptors. The multi-factor interaction network between immune cells was constructed and visualized by Cytoscape 3.7.2 (Fig. 3B, Supplementary Table 4).

### WGCNA analysis revealing key modules associated with breast cancer progression and patient survival

**Figure 4:**
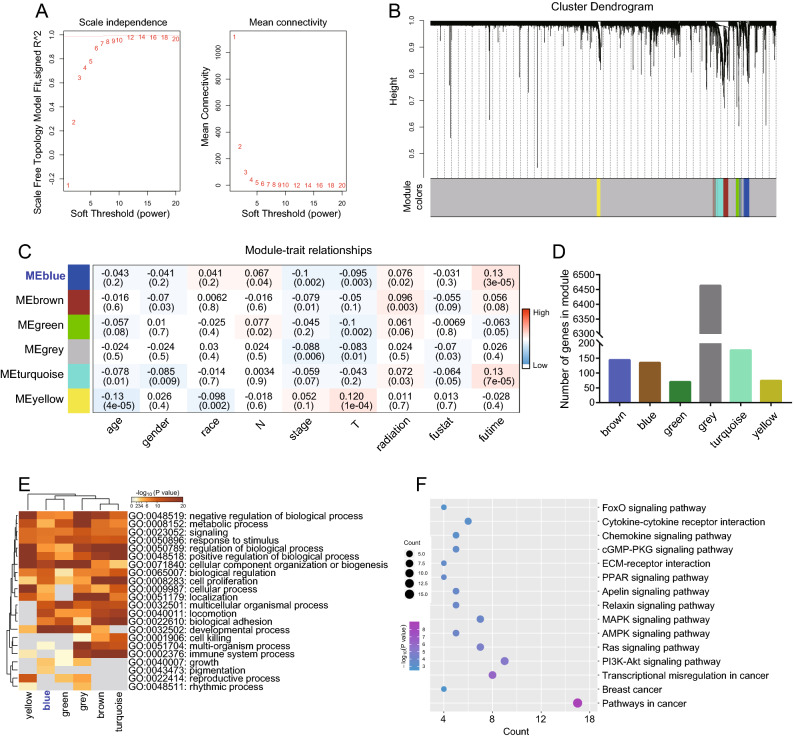
WGCNA analysis used to identify modules significantly associated with breast cancer prognosis. (**A**) Analysis of network topology for various soft-thresholding powers. (**B**) Module clustering analysis based on eigengenes. (**C**) Correlation analysis of each module and its traits. (**D**) Frequency statistical analysis of genes in each module. (**F**) Functional enrichment analysis of breast cancer prognosis-related modules. E: KEGG Enrichment Analysis of unregulated genes in MEblue module.

Based on the genes in the immune cell multi-factor interaction network, the grouping information of single-cell data (primary, metastatic, and triple-negative breast cancer) and the expression matrix, the co-expression network was constructed and WGCNA analysis was performed. The average-linkage hierarchical clustering method is used for gene cluster analysis. According to the standard of the hybrid dynamic shearing tree, the minimum number of genes (the soft threshold) for each gene network module is set. The results indicate that power = 18 is used as the threshold for subsequent analysis (Fig. 4A). After the eigengenes are calculated, the modules are subjected to cluster analysis, and finally 6 modules are obtained (Fig. 4B). Age, gender, race, N, the stage, T, radiotherapy, overall survival status, and overall survival time were used as indicators to screen the prognostic module with the highest correlation with breast cancer survival in different groups. The Pearson correlation coefficient between the ME of each module and the sample feature is calculated (the higher the module, the more important it is). The results showed the closest correlation between the ME blue module and the overall survival time of triple-negative breast cancer as well as the most significant difference (R = 0.13, P = 3e−05) (Fig. 4C). The contained genes are the main components representing the function and characteristics of the module. Meanwhile, the ME blue module incorporates 144 genes (Fig. 4D). These results indicate that ME blue may be a prognostic-related module in triple-negative breast cancer, playing an important role in predicting the disease progression and the overall patient survival. The GO enrichment analysis of the modular genes on the Metascape website shows that the ME blue module is mainly enriched in fields related to cell differentiation, movement, and proliferation, such as developmental process, locomotion, cell proliferation, multicellular organismal process, etc. (Fig. 4E) To further comprehend the mechanism of the ME blue module involved in the occurrence and progression of triple-negative breast cancer, the KOBAS website was used to conduct KEGG enrichment analysis on genes in the blue module. The results showed that the disordered genes in the ME blue module are mainly involved in the cancer pathway, cancer transcriptional mis-regulation, the PI3K-AKT signaling pathway, the Ras signaling pathway, the MAPK signaling pathway, cytokine-cytokine receptor interaction, and the AMPK signaling pathway (Fig. 4F).

### Univariate regression analysis combined with KM survival analysis to identify prognostic genes in ME-blue modules

**Figure 5:**
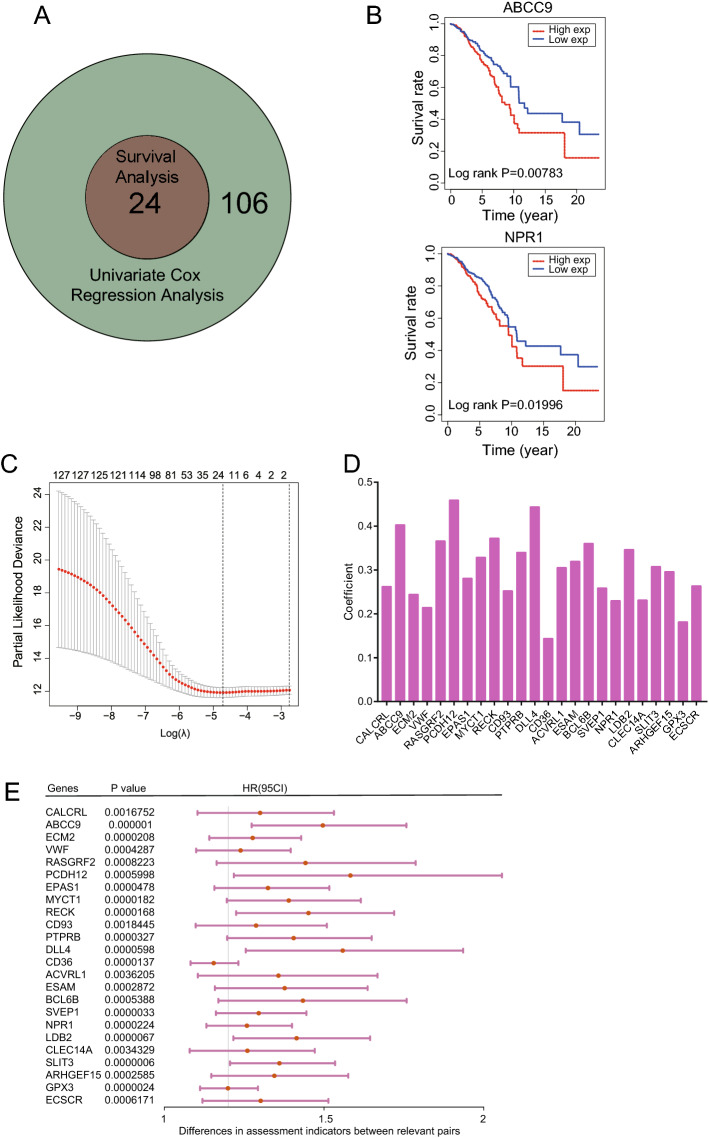
Univariate regression analysis and KM survival analysis of the BLUE module related to breast cancer prognosis. (**A**) Winn plots showing the intersection of significantly different genes in univariate regression analysis and KM population survival analysis. (**B**) KM survival curves of ABCC9 and NPR1. (**C**) Lasso dimensionality reduction analysis of 130 genes with significant differences in univariate analysis. (**D**) Correlation coefficient distribution of 24 intersection genes analyzed by univariate regression. (**E**) Visualization of univariate regression analysis of 24 intersection genes.

The coxph function in the survival package was used to analyze the relationship between the genes in the blue module and the overall survival (OS) of 1194 samples in the TCGA data, which downloaded from GDC and standardized by sklearn. Univariate regression analysis was performed on 144 genes in the blue module, and 130 genes with significant regression differences with P < 0.05 as the threshold (Supplementary Table 5) were obtained (Fig. 5A). Next, those 130 genes were subjected to lasso dimensionality reduction analysis, and the number of output genes was still 130 (Fig. 5B). Besides, the Kaplan–Meier method was used to analyze the overall survival of 130 genes in the blue module, and 24 genes were found to be in connection with prognosis of breast cancer (P < 0.05, Supplementary Table 6). This means that in the key module, 24 genes differ significantly in regression analysis and have prominent prognostic properties in survival analysis (Fig. 5A). After two genes (ABCC9, NPR1) were randomly selected for survival curve display (Fig. 5C), the correlation coefficients and the univariate regression analysis results of 24 genes were subsequently visualized (Fig. 5D,E).

### Multivariate regression analysis constructing a risk model of breast cancer prognosis-related genes

**Figure 6:**
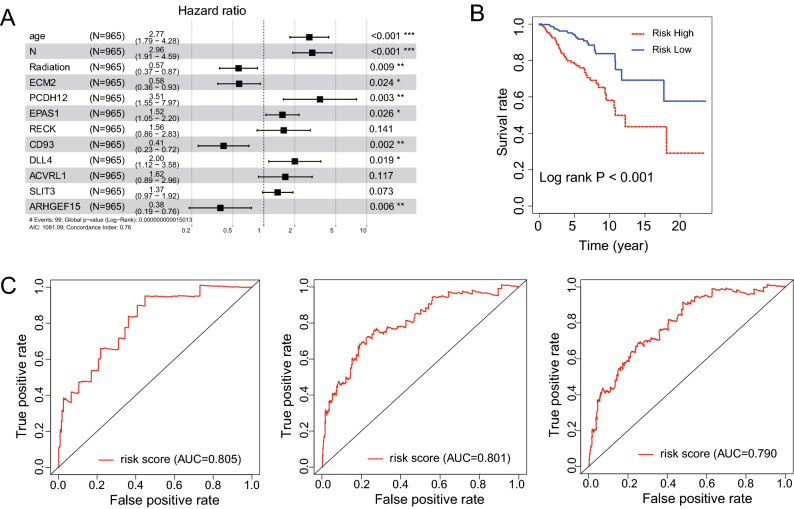
Efficiency evaluation of multivariate regression analysis and risk scoring. (**A**) The results of the multi-factor analysis displayed by the forest map. (**B**) Kaplan–Meier survival curves of high-risk and low-risk groups. (**C**) Time-dependent ROC applied to evaluate the accuracy of the model in predicting 1-, 3-, and 5-year survival.

**Figure 7:**
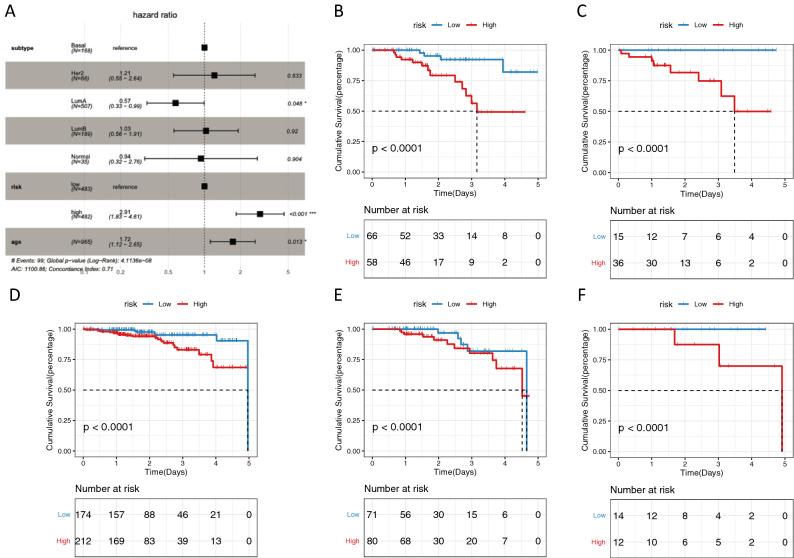
Efficiency evaluation of multivariate analysis and risk scoring. (**A**) The results of the multivariate analysis are displayed by forest map. (**B**–**F**) Cumulative survival of different molecular subtypes of breast cancer. (**B**: Basal-like, **C**: Her2-enriched, **D**: Luminal A, **E**: Luminal B, **F**: Normal-like.

To determine prognostic markers of breast cancer, the multivariate regression analysis of the 24 prognostic-related genes in the blue module was performed in combination with the clinical factors of TCGA (age, lymph node metastasis status (N0 vs N1 (excluding Nx)), T stage, radiotherapy or not, race, and the breast Cancer stage). The results suggest that the expression of ECM2, PCDH12, EPAS1, CD93, DLL4, and ARHGEF15 are significantly different in age, N (lymph node metastasis status), and whether radiotherapy is received or not (Fig. 6A). Subsequently, the sample data was scored and grouped in line with the median value of prognosis related gene expression. The Kaplan–Meier survival analysis of high-risk and low-risk group showed that the prognosis of high-risk one was poor (Fig. 6B, P < 0.001). The results of time-dependent ROC (the receiver operating characteristic) analysis found that the above-mentioned prognostic genes showed good predictive effects on the 1-year, 3-year, and 5-year survival of breast cancer (AUC was all greater than 0.75) (Fig. 6C). Among them, the AUC of the 3-year survival model was 0.801, which confirmed good accuracy of the prediction model (Generally, AUC >  = 0.7 is perceived as an effective predictor). The application of riskScore in different molecular subtypes of breast cancer is explored. Besides, molecular subtypes, risk scores and age are included in multivariate analysis. The results showed that risk score remained an independent prognostic factor for the molecular subtype and age (Fig. 7).

### Validation of a risk model constructed by genes related to the prognosis of breast cancer

**Figure 8:**
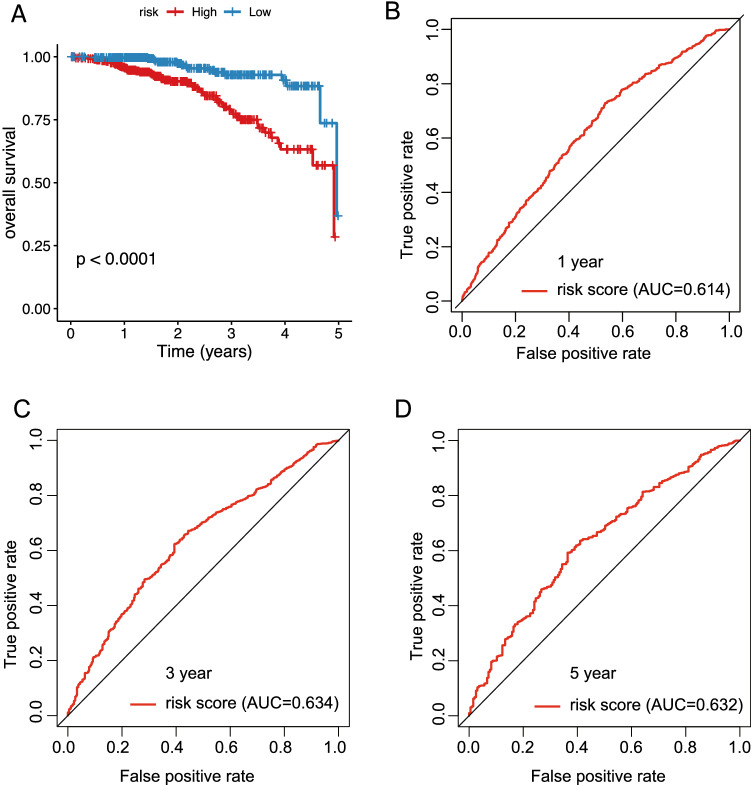
The accuracy of the breast cancer risk model constructed by prognostic related genes in predicting patient survival. (**A**) KM survival curves of high-risk and low-risk groups in ICGC data. (**B**) Accuracy of the time-dependent ROC assessment model in ICGC data for 1-year survival of breast cancer patients. (**C**) Accuracy of the time-dependent ROC assessment model in ICGC data for 3-year survival of breast cancer patients. (**D**) Accuracy of the time-dependent ROC assessment model in ICGC data for 5-year survival of breast cancer patients.

**Figure 9:**
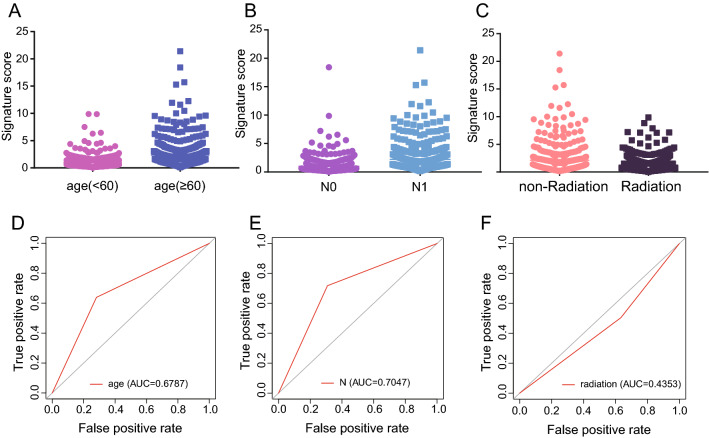
The accuracy of the breast cancer risk model constructed by prognostic related genes in clinicopathological indicators and prognosis (N0 indicates no lymph node metastasis and N1 lymph node metastasis). (**A**–**C**) Correlation between the risk score and clinical factors (AGE < 60 vs AGE > 60; N0 vs N1; Non-radiation vs Radiation). (**D**–**F**) Prognostic accuracy of risk scoring.

**Figure 10:**
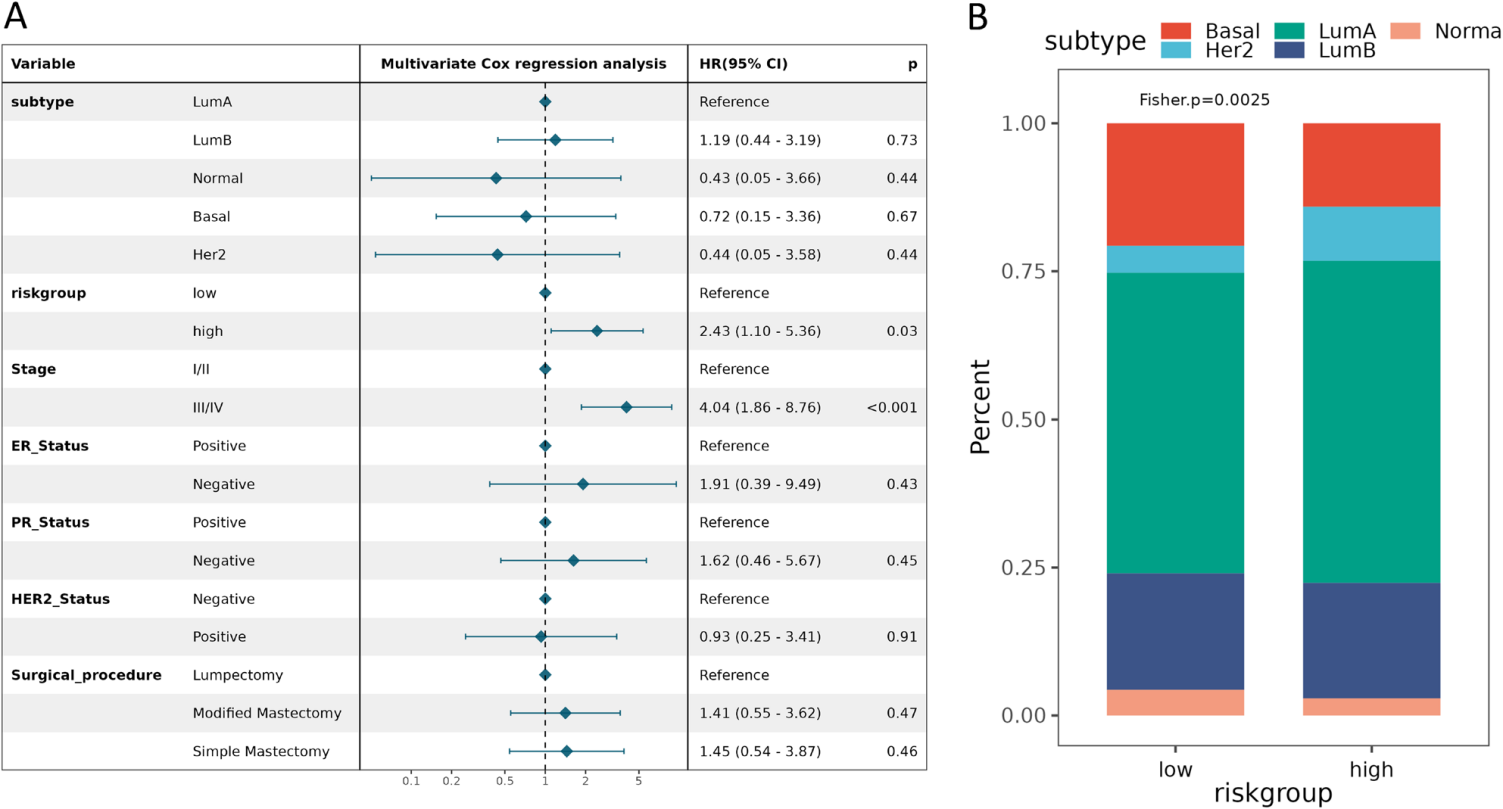
The multivariate regression analysis was performed in combination with the clinical factors and the distribution of molecular subtypes. (**A**) The multivariate regression analysis was performed in combination with the clinical factors. (**B**) The distribution of molecular subtypes in their high and low risk groups.

In this part of the analysis, breast cancer data from ICGC was used to validate the model constructed by the prognostic-related genes in the ME blue module, which downloaded from DCC and standardized by sklearn. The accuracy and validity of the above-mentioned model are verified by the use of 1542 sample data with overall survival time and overall survival status (Supplementary Table 7). The results indicate that the prognosis of high-risk group in the model is poor (P < 0.001, Fig. 8A). The time-dependent ROC results show that the AUC values for 1, 3 and 5 years are 0.614 (Fig. 8B), 0.634 (Fig. 8C), and 0.632 (Fig. 8D), respectively. Apart from that, according to our prognostic model, age, N lymph node metastasis and radiotherapy showed distinct differences in breast cancer samples with disparate risk scores (high-risk and low-risk) (Fig. 9A–C). Moreover, the verification results of ROC also confirmed that the AUC value of age, N lymph node metastasis was high, which indicated good accuracy of ROC (Fig. 9D–F). The multivariate regression analysis was performed in combination with the clinical factors (subtype, riskgroup, stage, ER status, PR status, Her2 status and surgical procedure). The results suggest that the riskgroup and stage are significantly different (P < 0.05, Fig. 10A). The distribution of molecular subtypes in their high and low risk groups was plotted (P = 0.0025, Fig. 10B). These results indicate that our model has practical application value in the prognosis and survival of breast cancer.

## Discussion

The heterogeneity of breast cancer is the main reason for treatment failure and recurrence. In recent years, the development of single-cell sequencing technology has deepen our comprehension of the heterogeneity of breast cancer. Cell types and specific gene expression characteristics in tumor tissues can be accurately distinguished by single-cell transcriptome. In reality, while breast cancer cells show significant heterogeneity, non-cancer cells, including fibroblasts, adipocytes, endothelial cells and various immune cells^17^, are the main content of heterogeneity in breast cancer^18,19^. Among non-cancer cells, the role of immune cells is particularly significant. The progression of breast cancer is characterized by increased immune cell infiltration in tumor parenchyma and stroma, including CD4^+^ and CD8^+^ granzyme B^+^ cytotoxic T cells, B cells, macrophages and dendritic cells^20^. In addition, tumor-infiltrating lymphocytes have been reported as a prognostic indicator of breast cancer chemotherapy response and patient survival^21^. This study found significant aggregation of NK cells in triple-negative breast cancer, a significant increase in the number of macrophages in primary breast cancer, and an increase in the proportion of B cells, T cells, and neutrophils in metastatic breast cancer. Although the high total number of NK cells reflects a good survival rate, the infiltration and activation of NK cells vary greatly among different sorts of breast cancer. The heterogeneity of NK cells and their actual roles in the microenvironment of breast cancer need to be further elucidated^22^. Tumor-associated macrophages (TAM) are the chief component of breast cancer microenvironment^23^. The increased density of macrophages in breast cancer tissues is related to the poor prognosis of patients, since macrophages are involved in the immune escape of breast cancer and the angiogenesis of tractable tumors^24^. In addition, B cells, T cells and neutrophils have all been reported to participate in the immune escape and metastasis of breast cancer^16,25,26^. These results indicate that our analysis results are correct and reasonable in terms of cell clusters and immune cell infiltration.

After cell clustering and annotation, a multi-factor interaction network of the ligand-receptor combined with transcription factors is constructed to discover modules significantly related to the prognosis of breast cancer. The results showed that the blue module had the highest correlation with the overall survival time of breast cancer (P = 3e−05). The functions of the blue module are mainly enriched in the aspects related to cell developmental, locomotion, and proliferation. The decrease of cell development has something to do with the poor differentiation level and cell stem characteristics of breast cancer; the enhancement of cancer cell motility is related to tumor invasion and metastasis; the disorder of cell proliferation is the basis of tumor tumorigenesis and progression. The enrichment results of KEGG showed that the primary signaling pathways for differential gene enrichment in the blue module include the pathway in cancer, transcriptional mis-regulation in cancer, the PI3K-AKT signaling pathway, the Ras signaling pathway, the MAPK signaling pathway, cytokine-cytokine receptor interaction, the MAPK signaling pathway, etc. PI3K-AKT, over-activated in most breast cancers, promotes the excessive proliferation of cancer cells through the mTOR complex^27^. For instance, the expression loss of the negative regulatory proteins PTEN and INPP4B (tumor suppressor genes) in the PI3K-AKT pathway is associated with the occurrence and progression of triple-negative breast cancer, and the loss of PTEN expression is found in more than half of TNBC patients^28^. In the Ras signaling pathway, activated Ras promotes the cell cycle and cell proliferation by recruiting Raf1 protein to initiate a kinase cascade to activate MAPK (ERK1/2) and transcription factors Fos and c-Jun^29,30^. In addition, activation of the Ras-MAPK pathway has been reported to facilitate TNBC immune escape^31^. p38MAPK signal was found to promote the invasion and metastasis of breast cancer by enhancing the epithelial-mesenchymal transition of cancer cells^32^. AMPK and its downstream mTOR are involved in the regulation of the material and energy metabolism of cancer cells 30,903,363. For example, AMPK-mediated lipid metabolism reprogramming promotes breast cancer cell proliferation and migration^33^. These results indicate that the cellular functions and signal pathways enriched in the blue module play a key part in the occurrence and progression of breast cancer. The validation results of the breast cancer prognosis model constructed by multivariate regression risk analysis showed that PCDH12, SLIT3, ACVRL1 and DLL4 genes are considerably different in the high-risk and low-risk breast cancer group, which can be used as risk factors for breast cancer prognosis.

Recent reports have found the association between high expression of PCDH12 and the high pathological grade of papillary renal cell carcinoma^34^. As a novel type of tumor suppressor gene, SLIT3 has been reported to play a role in breast, liver, lung, and colon cancer, and the promoter methylation of SLIT3 has been reported to be involved in tumor occurrence and progression^35,36^. ACVRL1 (the activin receptor like protein 1) encodes ALK1, which is a member of transforming growth factor—β receptor family and is associated with angiogenesis^37^. ACVRL1 expression can be used as a prognostic marker for patients with metastatic colorectal cancer who receive chemotherapy and bevacizumab^38^. DLL4, a major component of the Notch pathway, is reported to be highly expressed in breast cancer and associated with the advanced stage and distant metastasis of the patient^39^. These studies confirm the correctness of PCDH12, SLIT3, ACVRL1 and DLL4 genes as risk factors for breast cancer prognosis.

## Conclusion

MeBlue is a prognostic module in triple negative breast cancer. The expressions of PCDH12, SLIT3, ACVRL1 and DLL4 not merely relate to the type and proportion of immune cells, but also contribute to the prognosis of breast cancer.

## Data availability

The dataset supporting the conclusions of this article is available in GSE75688, GSE118389, TCGA-BRCA and ICGC(GSE75688:https://www.ncbi.nlm.nih.gov/geo /query/acc.cgi?acc = GSE75688.GSE118389:https://www.ncbi.nlm.nih.gov/geo/query/acc.cgi?acc=GSE118389.TCGA-BRCA:https://xenabrowser.net/datapages/?cohort=GDC%20TCGA%20Breast%20Cancer%20(BRCA)&removeHub=https%3A%2F%2Fxena.treehouse.gi.ucsc.edu%3A443.ICGC:https://xenabrowser.net/datapages/?cohort=ICGC%20(specimen%20centric)&removeHub=https%3A%2F%2Fxena.treehouse.gi.ucsc.edu%3A443).

## Supplementary Information

Supplementary Legends.

Supplementary Figure 1.

Supplementary Table 1.

Supplementary Table 2.

Supplementary Table 3.

Supplementary Table 4.

Supplementary Table 5.

Supplementary Table 6.

Supplementary Table 7.

Supplementary Table 8.
