# Supplementary material for: Pivotal models and biomarkers related to the prognosis of breast cancer based on the immune cell interaction network
Source: Sci Rep. 2022 Aug 11;12:13673. doi: 10.1038/s41598-022-17857-x (PMC9372165; doi:10.1038/s41598-022-17857-x)
Supplement: Supplementary file 1 — Supplementary Legends. [file 41598_2022_17857_MOESM1_ESM.docx]

Supplementary figure legends:Quality control and PCA analysis of two single-cell transcriptome sequencing data sets from the GEO database

A: The DimHeatmap function is used to analyze the main source of heterogeneity in the data set, and to determine the PC data for further downstream analysis. B: The Violin distribution form shows the distribution and proportion of the gene number (nFeature), the UMIs number(nCount), and the mitochondrial gene content (percent.mito) in the cell. C: The JackStrawPlot function is used to compare the p-value distribution and uniform distribution of each PC and determine the important PC (powerful and rich PC with low p-value). D: The ElbowPlot function sorts the principal components in terms of the variance percentage.

Supplementary table 1 legends: Cell marker of cellphoneDB database.

Supplementary table 2 legends: Marker for cell annotation

Supplementary table 3 legends: Result of ligand and receptor

Supplementary table 4 legends: The multi-factor interaction network between immune cells

Supplementary table 5 legends: Univariate regression analysis of HR for each gene

Supplementary table 6 legends: KM-survival. gene.0.05

Supplementary table 7 legends: Sample of Breast Cancer Susceptibility Gene
