# Supplementary figures and images for: Pivotal models and biomarkers related to the prognosis of breast cancer based on the immune cell interaction network
Source: Sci Rep. 2022 Aug 11;12:13673. doi: 10.1038/s41598-022-17857-x (PMC9372165; doi:10.1038/s41598-022-17857-x)

A

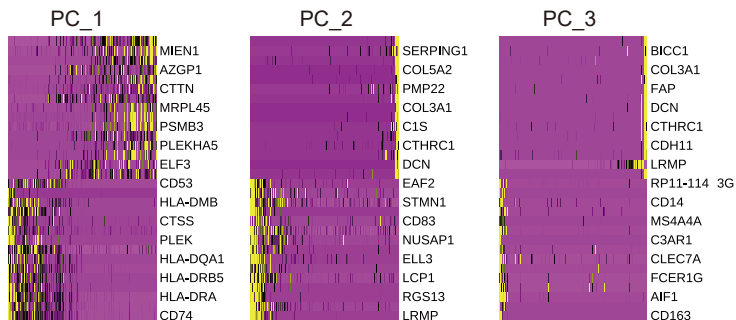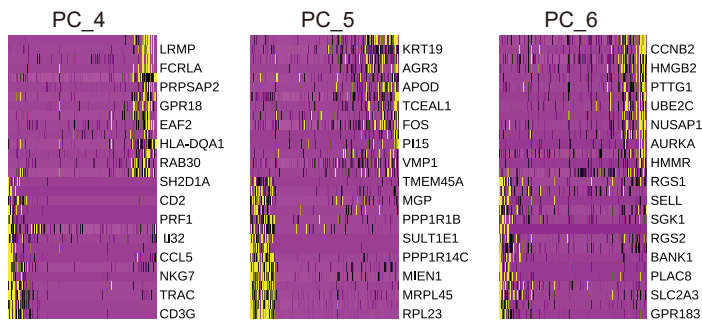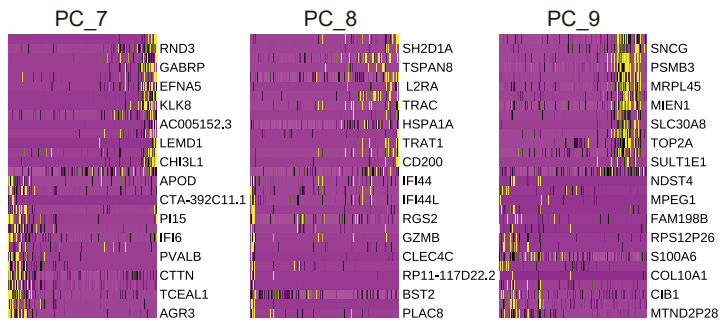

B

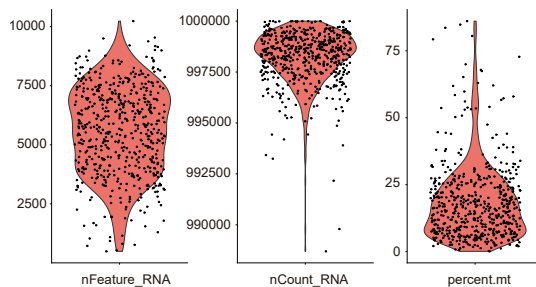

C

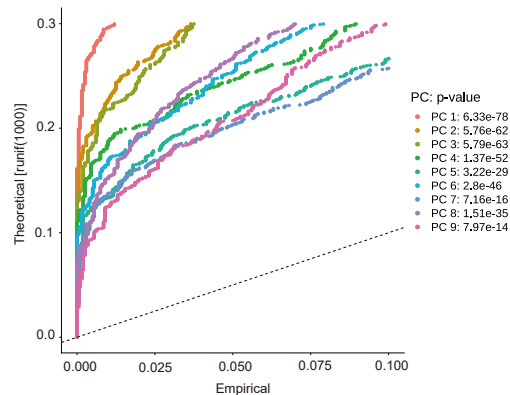

D

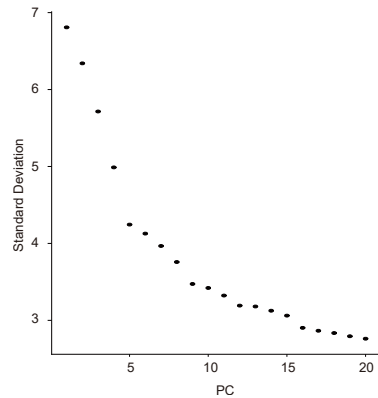

Supplement: Supplementary file 2 — Supplementary Figure 1. [file 41598_2022_17857_MOESM2_ESM.pdf]
